# Intra-operative device closure of perimembranous ventricular septal defect without cardiopulmonary bypass under guidance of trans-epicardial echocardiography: a single center experience

**DOI:** 10.1186/s13019-016-0483-2

**Published:** 2016-05-27

**Authors:** Yong Sun, Peng Zhu, Pengyu Zhou, Yilong Guo, Shao-Yi Zheng

**Affiliations:** Department of Cardiovascular Surgery, NanFang Hospital, Southern Medical University, GuangZhou, GuangDong People’s Republic of China

**Keywords:** Ventricular septal defects, Heart catheterization, Echocardiography, Minimally invasive

## Abstract

**Background:**

Intraoperative device closure of perimembranous ventricular septal defect(VSD) through a lower mini-sternotomy is safe, less invasive, and has excellent surgical and cosmetic outcomes. Our study is to evaluate the feasibility of closing VSD under guidance of trans-epicardial echocardiography.

**Methods:**

We reviewed the clinical course of 41 patients referred to our institution for minimally invasive closure of perimembranous VSD. The trans-epicardial echocardiography(TEE) was used to monitor the whole procedure to guide the positioning of device and evaluate the operative effect instantly after operation.

**Result:**

The procedure was successfully done in 38 patients(92.6 %) with mean age of 1.2 ± 1.5 years(range 0.5-6.1 years),mean weight of 10.78 ± 6.87 kg(range 5.2 ~ 26 kg) and VSD size of 5.1 ± 1.13 mm(range 5 ~ 10 mm). Three cases failed, including two cases whose guide-wires could not pass through VSDs and one case whose occluder could not repair VSD well. Three patients had tiny residual shunts because of the shifting of occluders. There were no major complications such as arrhythmia, valve regurgitation and the failure of occluder during follow-up(Mean 2.3 ± 1.2 years). TEE provided superior imaging of shapes and surrounding structures of the VSDs, and guide-wires passing through VSDs.

**Conclusions:**

Intraoperative device closure of perimembranous VSD through a lower mini-sternotomy without cardiopulmonary bypass appears to be a safe and effective procedure. The use of trans-epicardial echocardiography provides useful information for intraoperative device closure of VSD.

## Background

Perimembranous ventricular septal defect(VSD) is one of the most common congenital cardiac malformations.

Closure of VSD is routinely performed with cardiopulmonary bypass,or interventional transcatheter occlusion is performed for some selected patients [1, 2]. Traditional open heart surgery is safe with excellent outcomes, but this method with cardiopulmonary bypass is associated with postoperative complications [3–5]. The percutaneous device closure of perimembranous VSD is minimally invasive, but can be challenging because of low patient weight or poor vascular access, as well as multiple exposures to medical radiation [6, 7].

In recent years, intraoperative device closure of perimembranous VSD through a lower mini-sternotomy without cardiopulmonary has been introduced and applied clinically as an alternative treatment option [8–10]. Previous reports have demonstrated the safety and efficacy of this minimally invasive technique [9–11]. The transoesophageal or transthoracic echocardiography plays an important role in the process of selection of patients and closure of VSD [12]. However, little is known about the effectiveness of trans-epicardial echocardiography closure of perimembranous VSD during this procedure. Therefore, in the study, we reported the safety and efficacy of intraoperative device closure of perimenbranous VSD and evaluate the value of trans-epicardial echocardiography in the procedure.

## Methods

### Patients

Between May 2011 through June 2014, 41 patients with isolated perimembranous VSDs receiving the closure of VSD with an Amplatzer device (Shanghai Memory Alloy Company,Shanghai,China) using a lower mini-sternotomy approach under the guidance of trans-epicardial echocardiography at our hospital, were retrospectively reviewed. In our study, there were 16 females and 25 males, and the ages ranged from 0.5 to 6 years, the diameter of the VSDs ranged from 3 mm to 8.5 mm. Patients’ characteristics are listed in Table 1.

**Table 1:** Successfully ICOD patient characteristic (*N* = 38)

Variable	Values
age (years)	1.2 ± 1.5 (range 0.5 ~ 6)
Gender (F/M)	16/25
Weight (kg)	10.78 ± 6.87 (range 5 ~ 26)
Max-diameter of VSD (mm)	5.2 ± 1.13 (range 3 ~ 8)
Device size (mm)	6.8 ± 1.6 (range 5–10)
Hospital stay (days)	9 ± 3 (range, 6–12)
In-hospial complication	
Residual shunt (n)	3 (<2 mm)
Follow-up complication	
Residual shunt (n)	1 (<2 mm)
Follow-up time (years)	2.3 ± 1.2 (range, 0.5–3)

Patients were enrolled in this study for the following indications: (1) a hemodynamically left-to-right shunt; (2) the diameter of the Perimembranous VSD at the right-sided exit of at least 2 mm; (3) the subaortic rim(the distance from the defect to the aortic valve) of more than 1 mm. The following patients were excluded: (1) more than mild degree of aortic valve prolapse; (2) severe pulmonary hypertension; (3) newborn or young infants with a large VSD; (4) coexisting cardiac anomalies that need to be corrected at the same procedure.

Routine examinations included an electrocardiogram(ECG), a chest X-ray, and at least twice transthoracic echocardiography. Blood tests were done to exclude contraindications to antiplatelet therapy.

The informed consent about to participate in the study was obtained from the patient or guardian who was fully informed of the available treatment options. This technique was approved by the ethics committee of our hospital.

### Trans-epicardial echocardiography

Transthoracic echocardiography studies were performed to evaluate the position、size、shape and the surrounding rimes (especially the subaortic rim) of VSDs before the operations. After general anesthesia and a single-lumen tracheal intubation, patients were placed in a supine position. A continuous trans-epicardial echocardiography was used to monitor deployment of the devices. All patients were screened in the five-chamber view and left ventricular long-axis view to measure the diameter of the exit and entry (the left ventricular opening) of the defect. After deployment of the devices, the devices position and the integrity of the adjacent valve and the presence of residual shunt were assessed by trans-epicardial echocardiography repeatedly.

### Device and implantation technique

The technique of intraoperative device closure of perimembranous ventricular septal defect without cardiopulmonary bypass has been described in recent reports with some modifications [9, 11, 13]. There are three types of devices(Shanghai Shape Memory Alloy Co.Ltd, China) used in this study. An concentric occlude device with short-edged or wide-edged was about 2-4 mm in size larger than the connecting waist, while the superior part of left disc of an eccentric occlude device is as same as the diameter of VSD so as to prevent impingement of the aortic valve, and the inferior part with a metallic mark to indicate its location is 5.0 mm larger than the connecting waist. The right ventricular disc in the eccentric side is 2 mm larger than the waist. The delivery system includes a flexible guide-wire, double-lumen plastic delivery sheath and a loading sheath. Patients were placed in a surgical position under general anesthesia. A 3- to 4- cm lower partial median sternotomy and a pericardiotomy were performed [9]. The surface of right ventricular was exposed and heparinization was performed (1.0 mg/kg). Surgeon then determined the puncture site by slightly palpating the right ventricular free wall and locating the area of maximal thrill. A pledgetted 4-0 polypropylene mattress was placed at this location, and the free wall was punctured within the suture with a 20-G needle. A flexible hyperechogenic guide-wire was inserted and moved toward the exit of VSD under the guidance of trans-epicardial echocardiography, and then advanced through the defect, the needle was removed. A double lumen delivery sheath was introduced over the guide-wire under the guidance of trans-epicardial echocardiography, establishing the delivery pathway. During this process, the right ventricular orifice and guide-wire should be clearly continuously viewed through trans-epicardial echocardiography and imaging plane.

**Fig. 1:**
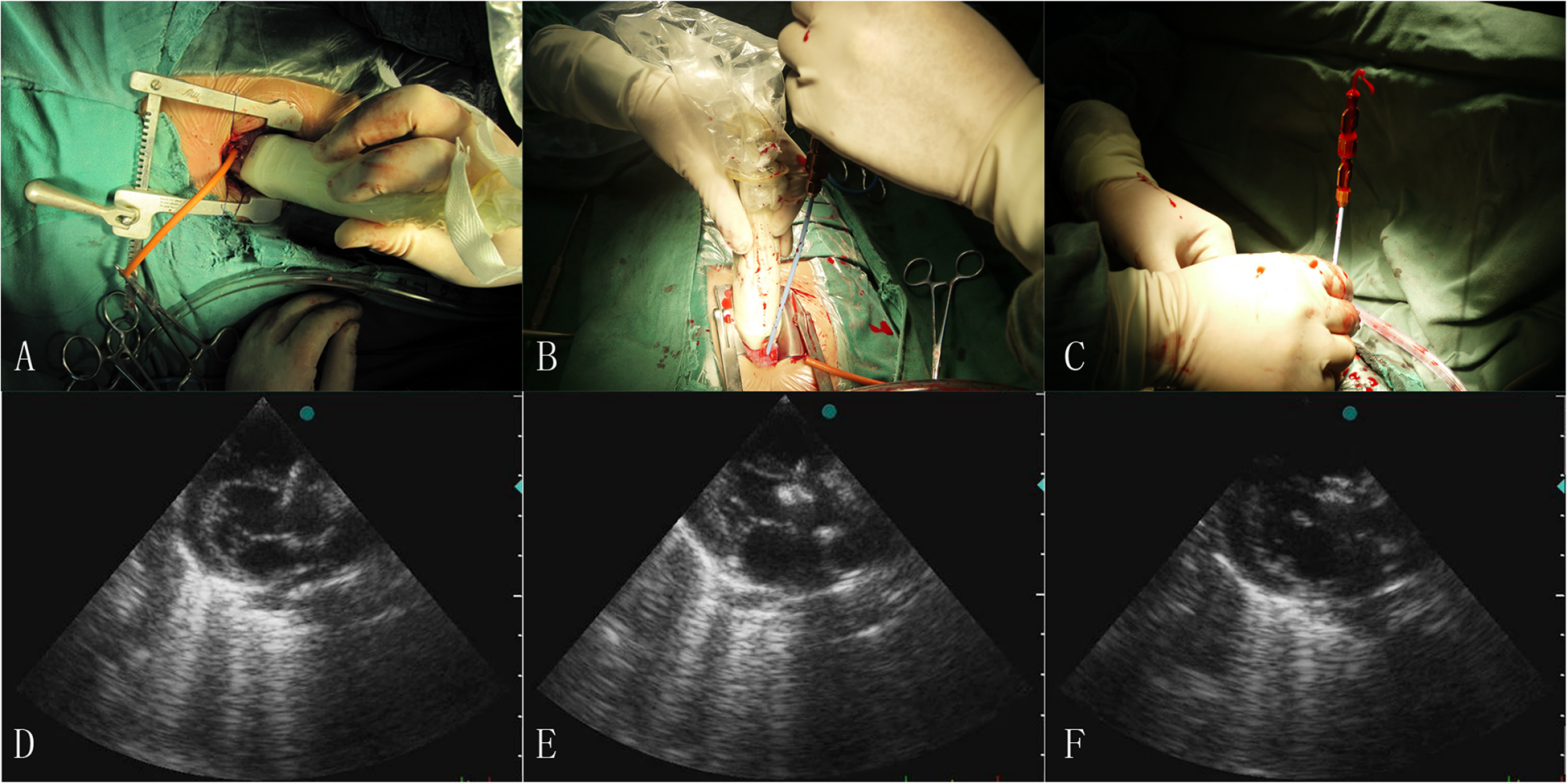
The steps of periventricular device closure of perimembranous VSD. (**a**) A trans-epicardial echocardiography view after a small stenotomy. (**b**) A surgeon handles the guide-wire under the guidance of trans-epicardial echocardiography. (**c**) Blood launching from the delivery sheath after it passed through the defect in to the left ventricle. (**d**) A right disc was deployed at first step. (**e**) A device was completely deployed and the delivery sheath will be kept for 5 min. (**f**) A device was successfully deployed without any complications

The occlude device was delivered along the sheath. Holding the delivery sheath and then the left ventricular disc was deployed by pushing the cable forward. After deployment of left ventricular disc, the delivery sheath and cable were slowly and simultaneously withdrawn until the left disc was snugly against the ventricular septum wall, and the waist as well as the right ventricular disc was released. After the occlude device was fully deployed, surgeon should test its stability by using a to-and-fro motion maneuver repeatedly before the delivery system was removed. In order to ensure that the eccentric occluder was released in an appropriate position, the occlude device was adjusted until the eccentric side of the disc was opposite to the aortic valve and faced towards the heart apex after the marker of eccentric side were visualized by trans-epicardial echocardiography (Fig. 1).

Because the perimembranous VSD is closed to the aortic valve, valve regurgitation or right ventricular outflow tract obstruction should be avoided. First, the occluder could be easily retracted and redeployed in case of inappropriate released position of the occlude device. Second, it was crucial for the operator to be very cautious during releasing the occlude device.

Trans-epicardial echocardiography study was then performed to assess positioning of the device, residual shunt, and device malposition as well as any obstruction or regurgitation of the valve induced by device [9, 12]. The degree of residual shunt was assessed by measuring width of the color jet [12]. Trans-epicardial echocardiography was kept for 15 min to observe the position of device before the sheath was withdrawn. The purse-string was snugly tied and heparin was neutralized after the sheath was then withdrawn. After a drainage tube was placed in the pericardium, the mini-thoracotomy was closed.

### Follow-up

Prophylactic antibiotics were administrated before the procedure and last for 24 h for prophylaxis of bacterial endocarditis. After operation, low dose oral aspirin (3 ~ 4 mg/kg/day) was given to the patients and was continued for at least six months.

The follow-up protocol included an ECG and TTE that were scheduled at discharge, 3, 6, 12 months after the procedure. The position and stability of the device, residual shunt and procedure-induced valve complication were carefully checked during the follow-up examination.

### Data analysis

Datas are expressed as median or mean ± SD and range.

## Results

### Intraoperative and Early postoperative period

A total of 38 patients(92.8 %) underwent intraoperative device closure of perimembranous VSD, and the procedure was converted to conventional open repair under cardiopulmonary bypass in the remaining 3 patients(7.2 %). The sizes of the exits of perimenbranous VSDs ranged from 3.0 mm to 8.5 mm(5.45 ± 1.27 mm), and the sizes of occluders ranged from 5 mm to 10 mm. In the successful VSD cases, the concentric occlude devices with wide-edged were implanted in 27 patients(71.1 %) and the eccentric occlude devices were implanted in the remaining patients. All the patients did not require transfusion of blood products.

New trivial or mild residual shunt was detected in 3 patients(8 %) by intraoperative trans-epicardial echocardiography. No worsening tricuspid valve regurgitation was observed in those with existing tricuspid regurgitation before surgery, and no more than mild degree of regurgitation was found in this study. No obvious aortic valve regurgitation was detected during the procedure. No incomplete bundle branch block or complete atrioventricular block was detected during the procedure or before discharge.

All successful patients were extubated within 2 h after the procedure, and were discharged in 5 days.

### Conversion to open repair

Three patients had unsuccessful procedures, which were all converted to conventional open repair with cardiopulmonary bypass. The guide-wire failed to cross the VSD in 2 patients after we moved guide-wire repeatedly and tried different puncture areas. During the surgery, membranous aneurysms and the size of VSD small than 3 mm were found in 1 patient, and the entry on the LV side and the exit of membranous aneurysm on the RV side were not in the same axis in another patient. A 5-mm occlude device was successfully implanted in a 3-mm membranous aneurysm VSD under the guidance of trans-epicardial echocardiography, but it fell off in 1 day after operation. During open surgery, weak and friable wall of the membranous aneurysm VSD with more than 10-mm size on the LV side was found.

### Follow-up results

Follow-up period of all successful patients ranged from 0.5 to 3 years(mean2.3 ± 1.2 years). The short and mid-term follow-up results were available by electrocardiography and trans-epicardial echocardiography, 38 patients for 1 month、3 months、and 6 months,27 patients for 1 year, and 19 patients for 2 years and 12 patients for more than 3 years. Symptoms were much improved or totally resolved in all symptomatic patients. Small shunts were found in 3 patients immediately after operation. After 6 months follow-up for these 3 patients, shunts disappeared in 2 patients and remaining shunt decreased in another patient. No occlude device dislocation, thrombosis, complete heart block, new aortic regurgitation and other major complications were found during the follow-up period.

## Discussion

Intraoperative device closure of perimembranous VSDs without cardiopulmonary bypass was first reported by Amin and cleagus [5], and has become an accepted modality of treatment in many cardiac centers [6, 9, 13]. This approach has many important advantages compared with conventional surgery, though surgical closure has been considered as a standard technique with optimal long-term result for permembranous VSD for a long time. This technique has many advantages, including no potential complications of CPB, surgical trauma, no need for blood transfusion, shorter time in ICU, smaller incision, less pain for patients and faster recovery and cosmetic result. In recent years, percutaneous closure perimembranous VSD as an accepted alternative treatment option to cardiac surgery [14, 15]. Details of this technique and its encouraging mild-term outcomes have been extensively described in several studies [16]. However, this method is associated with many disadvantages including the limitation of vascular access, the complexity of technique, perforation of the heart, tricuspid valve damage. Meanwhile, residual shunt, the malposition and failure of device are common [17].

Additionally, this technique needs more advanced and expensive equipment [18]. Compared with the percutaneous procedure, the advantages of periventricular technique seem to be obvious. First, there is no vascular access injury from sheath insertion or guide-wire, and this technique is not constrained by patients age and weight compared with transcatheter intervention. Second, the device can be easily controlled and adjusted during the procedure, because periventricular technique has excellent manipulability of a short and straight sheath and guide-wire, the reliability of testing the stability of device for a to-and-fro maneuver. With the trans-apeicardial echocardiography imaging, this technique is easier and safer. In case of the occlude device dropping off or inappropriate selection of cases for device closure, full sternotomy can be more quickly performed to close the VSD under CPB, because the procedure is performed by the same surgeon in the operating room. Third, there is no exposure to radiation especially for children, and more advanced equipment is not needed in periventricular technique.

The key point of periventricular VSD closure is that sheath and guide-wire should pass through the VSD under echocardiography imaging [13, 19]. Transoesophageal echocardiography, which was done immediately before, during and after deployment of the occlude device, has been considered as a standard technique for this procedure [12]. Once the device is released, a repeat transoesophageal echocardiography is performed to assess the position of device, the presence of residual shunt and aortic valve regurgitation. Trans-epicardial echocardiography can provide useful information for intraoperative device closure of perimembranous VSD. However, little is known about the effectiveness of periventricular close of perimembranous VSD using trans-epicardial echocardiography. The site, size, and rims of the defect, the suitability for device, and guiding the guide-wire and sheath to pass through the VSD can be detected by trans-epicardial echocardiography with clear imaging during the procedure. The reference position, which is also the region of interest, was based on the whole structure of the perimembranous VSD. We also found that trans-epicardial echocardiography could provide more clear view of vessels when surgeons are trying to pass through the perimembranous VSD with the guide-wire and sheath. Compared with transoesophageal echocardiography, the advantages of trans-epical echocardiography are no need of a transoesophageal probe and no operation trauma.

Several factors contribute to the success of the intraoperative device closure of perimembranous VSD [13, 19, 20]. First, the size of perimembranous VSD is small (<3 mm) and the jet of VSD directs toward the tricuspid valve, therefore, it will be very difficult for sheath to pass through VSD with a smaller diameter of defect, which may result in the failure of implantation. Second, in our experience, the occlude device closure may fail if the sensitive finger could not feel the murmur on the surface of right ventricular free wall after a small lower sternotomy. Third, the perimembranous aneurysm VSD usually has soft rim. According to our experience, it is recommended that in such case with VSD of a soft rim, the size of the occlude device should be 4 or 5 mm larger than the maximum diameter of VSD. And in order to avoid the failure of device and the presence of residual shunt, operators should adjust device repeatedly until it is released.

In one perimembranous aneurysm VSD patient, no residual shunt or device malposition was observed after procedure, however, the device failed in 1 day after operation because of the weak and friable wall of the membranous aneurysm and malposition of occluder.

In our study, only one occlude device failed in 1 day after operation, and no complete AVB or left bundle branch block occurred, aortic valve insufficiency or device embolization was observed in this group. As mentioned earlier, compared with the transcatheter intervention, only a shorter delivery pathway is required and no sheath or guide-wire passes through the atrial-ventricular valve in this technique. The eccentric side of the occlude device can be easily manipulated by the operators, and the device can be adjusted to face the heart apex to avoid the risk of aortic valve regurgitation. Therefore, the incidence of complications related to the catheter-placed occlude devices was very low in this group. Because the VSDs are small in diameter and the jet flow may face towards the sheath, and there are many other structures(tricuspid valve and its tendons) around the perimembranous VSDs, it will be difficult for beginners to pass the sheath through the VSDs. In our experience, a real-time trans-epicardial echocardiography monitoring by experienced cardiologists and the handling the guide-wire and sheath flexibly by surgeons can solve this problem. The VSD repair under CPB could be finished by cardiac surgeons themselves, and after 10 to 20 operations, the entire procedure was simplified.

### Limitations

All the findings of this study should be interpreted with caution considering the following limitations. First, although the techniques of the periventricular closure of perimembranous VSD under trans-epicardial echocardiography guiding seem to be safe in the mid-term follow-up, more experience and long-time follow-up results are necessary to assess the actual effectiveness and safety of this technique as an alternative to conventional surgery and transcatheter intervention. Second, this study is not a prospective randomized study, therefore, despite of the excellent results, we just draw the conclusion with past experiences and results of this technique.

## Conclusions

In conclusion, as far as we know, this is the first published report of mid-term follow-up outcome of intraoperative device closure of perimembranous VSD under trans-epicardial echocardiography. Intraoperative device closure of perimembranous VSD is minimally invasive, safe, cosmetic and feasible for many patients, and as well as for infants. A small lower sternotomy and trans-epicardial echocardiography make it unlimited to the age and weight of patients.

## Abbreviations

AR, Aortic regurgitation; AVB, Atrioventricular block; CPB, Cardiopulmonary bypass; TTE, Transthoracic echocardiography; VSD, Ventricular septal defect.
